# Multi-Level-Phase Deep Learning Using Divide-and-Conquer for Scaffolding Safety

**DOI:** 10.3390/ijerph17072391

**Published:** 2020-04-01

**Authors:** Sayan Sakhakarmi, Jee Woong Park

**Affiliations:** Department of Civil and Environmental Engineering and Construction, University of Nevada, Las Vegas, NV 89154, USA; sayan.sakhakarmi@unlv.edu

**Keywords:** construction safety, risk, scaffold, deep learning, divide-and-conquer

## Abstract

A traditional structural analysis of scaffolding structures requires loading conditions that are only possible during design, but not in operation. Thus, this study proposes a method that can be used during operation to make an automated safety prediction for scaffolds. It implements a divide-and-conquer technique with deep learning. As a test scaffolding, a four-bay, three-story scaffold model was used. Analysis of the model led to 1411 unique safety cases for the model. To apply deep learning, a test simulation generated 1,540,000 datasets for pre-training, and an additional 141,100 datasets for testing purposes. The cases were then sub-divided into 18 categories based on failure modes at both global and local levels, along with a combination of member failures. Accordingly, the divide-and-conquer technique was applied to the 18 categories, each of which were pre-trained by a neural network. For the test datasets, the overall accuracy was 99%. The prediction model showed that 82.78% of the 1411 safety cases showed 100% accuracy for the test datasets, which contributed to the high accuracy. In addition, the higher values of precision, recall, and F1 score for the majority of the safety cases indicate good performance of the model, and a significant improvement compared with past research conducted on simpler cases. Specifically, the method demonstrated improved performance with respect to accuracy and the number of classifications. Thus, the results suggest that the methodology could be reliably applied for the safety assessment of scaffolding systems that are more complex than systems tested in past studies. Furthermore, the implemented methodology can easily be replicated for other classification problems.

## 1. Introduction

### 1.1. Background

The majority of construction sites use scaffolds as temporary structures to support workers and construction materials. Scaffolds possess various safety hazards, such as workers falling from a height, being struck by equipment or materials, or being electrocuted, as well as the scaffold collapsing [1]. These potential hazards endanger the lives of 65% of all construction laborers working on scaffolds in the United States [2]. Despite regular safety inspections and safety training at construction sites, many laborers are exposed to fatal accidents every year [2]. To reduce this safety problem, researchers have investigated various methods to improve worker safety in the early stages of construction through the automation of scaffolding structure design [3,4], as well as planning [5,6] and the application of building information modeling [5,7,8,9]. Although these studies have advanced steps forward in automated safety planning and design, they are not applicable during the construction stage. 

To assist in the monitoring of safety problems during construction, a few research studies have sought real-time monitoring approaches [10,11,12,13]. Xue et al. [10] proposed the use of radio frequency identification (RFID), together with scaffold safety management, in order to warn safety managers in real-time. Yuan et al. [11] developed a cyber–physical system, which integrates the temporary structure with its virtual model in order to automate potential hazard detection. Jung et al. [12] explored the capability of sequential image processing to automate the possible failure detection of temporary structures. Cho et al. [13] demonstrated the applicability of machine learning (ML) to automate the classification of scaffold safety conditions, using the strain measurements of the scaffold members. However, these efforts to automate scaffold safety assessments are based on a limited number of safety conditions, which are significantly simpler with respect to the mode of failure corresponding to scaffolds used at most construction sites [11,12,13]. As past investigations have identified hazards by checking against threshold values [11], or accounting for only a small number of failure cases [11,12,13] associated with structural configuration, there may be difficulty in establishing their applicability for generic, temporary structures and addressing their potential risks.

On actual construction sites, small scaffolds are only used for minor site activities, and most sites use multi-bay and multi-story scaffolds. Such structures typically involve many modes of potential failure, and the identification of local member failures, which increase significantly in number, becomes critical in properly identifying the potential failures of the system in advance, in order to take precautionary measures. Despite the importance of identifying such failures, no research has adequately covered this challenge, except for the authors’ previous study [14], which introduced the need to consider such local-level failures, but only considered individual member, local-level failures when classifying the scaffold failure cases. Accordingly, the authors performed a simple classification of 23 failure modes for a scaffold, and investigated the effect of modifying the number of strain features on the prediction accuracy of failure cases. Unfortunately, the identification of all such failures entails complexity in analysis, and inevitably results in a lower prediction accuracy if the same approaches that were employed with small scaffolds are used. This challenge can be further exacerbated, resulting in additional modes of failure, as a result of construction activities (e.g., changes with work location, positions of equipment and workers, placement of construction materials, etc.), which continuously change throughout the construction period. Without confirming the capability of the automated approaches on such complex cases, past research cannot be translated into practice. Therefore, there is a need for an efficient safety assessment system that is capable of classifying many modes of failure cases during the safety assessment.

Cho et al. [13] demonstrated real-time integration of an ML technique—a support vector machine (SVM)—with a real-time strain sensing system, at 97.66% accuracy. This prototype demonstration presented the possibility of ML and a real-time sensing application for the realization of an automated safety monitoring system. Despite its achievement with high accuracy, this study suffered a few challenges: (1) a small number of safety categories, as the prototype study considered only four classifications; (2) redundant classifications; and (3) the inability to make predictions in some cases. Based on these concerns, it is reasonable to expect that an SVM will encounter more unreliable cases with the large type of safety classification that this study attempts to explore. To overcome this problem, there is a clear need for a more in-depth investigation of advanced algorithmic approaches, as well as the relationship between the safety conditions and strain measurement data. 

### 1.2. Application of Deep Learning 

The consideration of local or global level failures, along with the combination of member failure modes, depends on the level of detail required during the safety assessment. As one accounts for local level failures, as well as the combination of member failure modes, there is a natural increase in the number of failure cases. This increase poses a difficulty in the precise monitoring of scaffolding safety conditions. In the aspect of ML classification, the challenge associated with this difficulty is the required number of classifications. Scaffold safety prediction with ML requires a large database for training and testing purposes. Deep learning, because of its flexibility with large databases and its capability to handle a large number of classifications, has received significant attention from construction researchers [15,16,17,18,19,20,21,22,23,24,25,26]. For example, with safety applications, researchers have used deep learning to identify non-certified workers on construction sites, in order to prevent safety hazards [15]; to identify workers, equipment, and materials [16,17,18]; to identify unsafe worker behavior, in order to prevent accidents [19,20,21,22]; and to detect guardrails [23] and cracks [24,25,26]. However, all of these studies are related to computer vision, while the authors in this study intend to automate the prediction of scaffold safety conditions using numerical strain measurement values. Therefore, this research uses a deep learning technique, a neural network (NN), as a part of its methodological development. 

### 1.3. Divide-and-Conquer Method

One of the problems associated with large-scale classification is the large number of classes it includes [27]. Classification of a large number of classes operates well with enough features and datasets. However, it is inefficient in a practical sense to collect sensor data from many different points of a scaffold, as it would be very expensive to attach many sensors to a temporary structure every time it is built for construction activities. This inevitably results in a limited number of features, which the authors have attempted to overcome by applying the divide-and-conquer technique. This technique has demonstrated performance improvement in multi-class classification, with both NN and SVM classifications [27,28,29].

The divide-and-conquer technique utilizes a hierarchical structure to solve problems involving large datasets with a large number of classes. Generally, this technique is widely used to solve speech and text recognition problems. Fritsch and Finke [28] demonstrated the implementation of this technique in speech recognition, which requires the classification of thousands of classes with a large training dataset. The implementation of the divide-and-conquer method involves three major steps: breaking the main (large) problem into small problem sets; training the classifiers for the small problem sets; and finally, combining the classifiers to solve the main problem. This technique will be particularly useful for the current research problem, because it allows the authors to effectively use a limited number of features on smaller problem sets. Because of the aforementioned practical limitations of strain data measurements, this technique becomes key in turning a difficult problem, with large classifications that have a small number of features, into a relatively easier problem by including smaller classifications with the same features. Therefore, this study explores the divide-and-conquer approach to overcome the challenge of predicting a large number of failure cases with limited strain features. 

## 2. Research Objective and Scope 

The objectives of this research are to improve scaffold safety monitoring methods and overcome limitations presented in past studies—mainly, the classification of a small number of failure cases for small scaffolds [11,12,13]. To accomplish these, this research designs a method to detect both the global and local member failures of scaffolding structures, as well as complex combinations of member failures. 

The scope of this research is bound with 1,540,000 computer-simulated datasets, 1411 identified failure modes, and a hybrid analytical methodology. The presented research is built based on the scientific evidence of a previous study [13], which is that real-time strain data can be fed into an ML-based analysis. However, the prior research [13] focused on the capability of real-time integration on relatively simple systems (e.g., only four failure modes). Therefore, the scope of this research is to explore a method that can increase the analysis capability of such a system for a larger number of analyses; the authors present 1411 failure modes to detect, while past studies have addressed fewer. 

To solve this large-scale classification problem, this research will focus on exploring the capability of a divide-and-conquer technique combined with deep learning, as demonstrated by other studies [27,28,29], through computer simulation. The authors consider only 20 sensor data points, one per column, for the tested scaffolding system; having more sensors will consequently improve the accuracy of the system by nature, but it may become impractical to use many sensors on temporary structures at construction sites. Twenty is a reasonable number of sensors to deploy, especially for the size of the studied scaffold. The proposed methodology is explored particularly in order to contribute to improvement in automating the prediction of scaffolding conditions. Finally, this research will produce an output that can serve to identify unsafe scaffold sections in an active construction zone based on strain measurements.

## 3. Materials and Methods 

**Figure 1:**
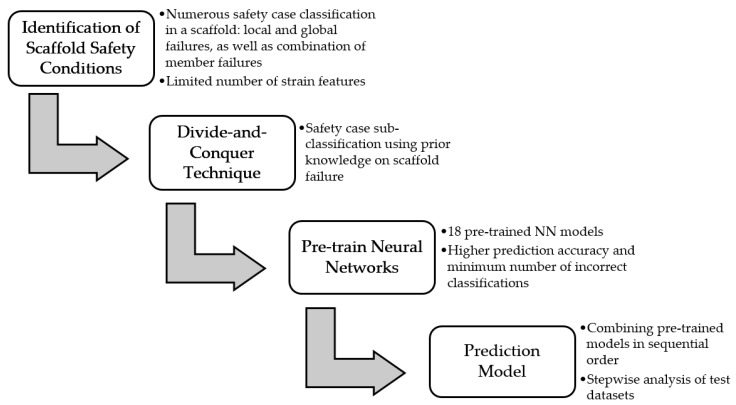
General approach.

The general approach of this study was to apply a divide-and-conquer technique with deep learning to automate scaffold safety predictions from numerous safety cases. The prediction is based on the strain measurements of individual scaffold members. Figure 1 illustrates the steps in the proposed methodology. The authors first identify the problem in a number of categories for quantification purposes. Then, to alleviate the problem associated with a large number of classifications based on a limited number of features, a divide-and-conquer technique is applied. This technique subdivides the entire set of problems into subsets, which are grouped into similarity-based categories. NN models are then applied, and their training parameters are obtained at each level of classification. These pre-trained NN models are compiled together to form an integrated NN model for follow-up prediction. The following sections explain the approach in detail.

### 3.1. Identification of Scaffold Safety Conditions

For safe working conditions, a scaffold must be structurally safe; however, there are many factors that make scaffolds unsafe. Based on the mode of scaffold failure, this study categorized the unsafe cases into over-turning, uneven settlement, and overloading cases [13]. Furthermore, in order to ensure the inclusion of all potential failure cases, both local and global failures, as well as a combination of different member failure cases, were considered. Therefore, the three unsafe modes of scaffold failure could be further classified into more specific failure modes, which converts the problem into a fine scale problem with significant complexity in classification. The over-turning failure of a scaffold is a global failure, resulting from sidewise movement; therefore, over-turning failure is sub-classified into failure in longitudinal (*X*) and transverse (*Y*) directions, based on the direction of sidewise movement. Similarly, uneven settlement and overloading failure cases may result from the local failures of the scaffold members in various combinations. Therefore, these two failure cases are sub-classified on the basis of the different numbers of column failures and their combinations during the load application. For example, a scaffold with *n* number of vertical sections may have overloading failure due to overloading on a single vertical section, or a combination of two to *n* vertical sections at a time. 

**Figure 2:**
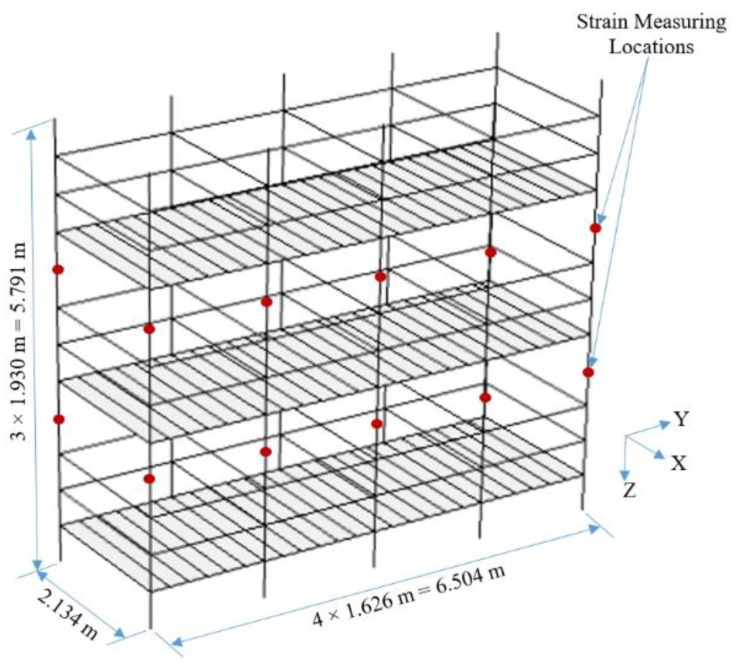
Scaffold model.

To visualize all such possible failure cases, the authors selected a four-bay, three-story scaffold model for this study. In the model, the pipes were modeled using beam-column elements, and the planks were modelled using shell elements. The model was designed using a commercial package called COMSOL Multi-physics [30], with a reference to a past study by Cho et al. [4] that validated a scaffold modeling technique to reflect actual structural behavior. The model design followed the Occupational Safety and Health Administration (OSHA) scaffold specifications [31]. Figure 2 illustrates the scaffold model, in which each bay measures 2.134 m (7.00 ft) × 1.626 m (5.33 ft), while the height of each floor is 1.930 m (6.33 ft). The figure also shows the locations of the strain sensing data points on each of the vertical sections. There are 20 strain measurement points on the scaffold model, one each at the top and bottom vertical members, to record the members’ strain behaviors on various loading cases. For this scaffold model, the authors identified a total of 1411 different safety conditions. 

The model is structurally analyzed to create a database of strain measurements at the allocated points for various loadings. The authors applied a number of random load combinations, as shown in Table 1, to generate varying loading conditions for realistic training data preparation. For each of the 1411 cases, COMSOL was used to generate 1000 datasets to be used for ML. Each dataset was comprised of 20 strain measurements corresponding to the strain measurement points, as shown in Table 2. As such, the database was generated with 1,411,000 total datasets, which is equivalent to 1000 datasets for each of the 1411 safety classifications. This database was used for training and testing the pre-trained NN models, in order to obtain the required pre-trained NN model parameters. Similarly, a new database consisting of 141,100 datasets (i.e., 100 datasets for each of the safety classifications) was generated for testing the performance of the integrated NN model.

### 3.2. Divide-and-Conquer Technique

**Figure 3:**
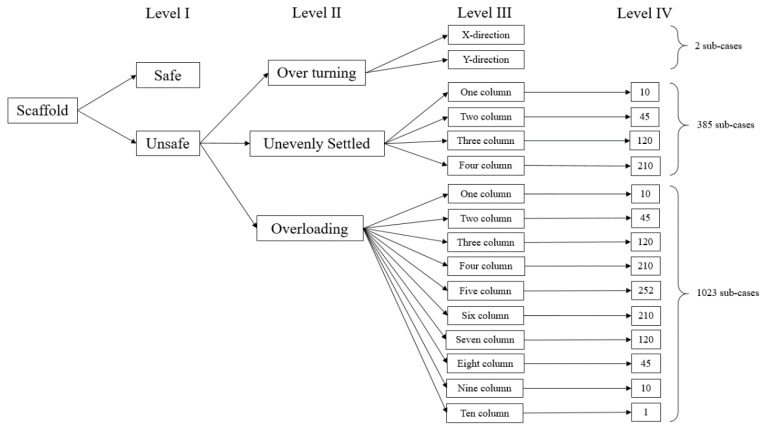
Hierarchical classification of safety conditions based on the divide-and-conquer technique.

For divide-and-conquer technique implementation, the first step is to simplify the multi-class classification problem by dividing it into smaller problems. A basis for the division of a larger problem into a hierarchical classification is to utilize prior knowledge about the existing problem [28]. In this case, the authors implemented their prior knowledge on different modes of scaffold failures to obtain sub-classifications. First, the overall problem was classified into safe and unsafe cases, then the unsafe cases were classified into three modes of failure: over-turning, uneven settlement, and overloading. These were then further categorized, considering local and global failures modes, as well as combinations of different member failures. Overturning failure was divided into two sub-cases. Depending on the number of columns that failed, the unevenly settled and overloading failures were divided into four and ten classes, respectively. Considering combinations of member failures, these classes were further divided into 385 and 1023 sub-cases, respectively, for the unevenly settled and overloading scenarios. Thus, this approach resulted in four different levels of classification, as illustrated in Figure 3, in which the safe condition is identified in Level I, and unsafe categories are identified in Levels III and IV. All safety conditions are numerically indexed from 1 to 1411, where Index 1 refers to a safe scaffold and Index 1411 refers to an overloaded scaffold case, with a failure of ten columns at a time.

**Figure 4:**
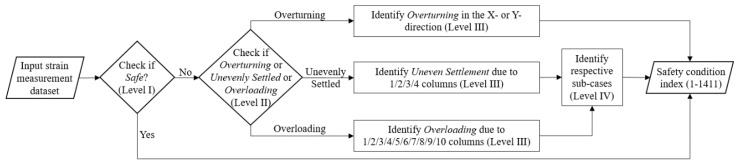
Prediction model flowchart.

Following the hierarchical classification model, the final model resulting from this approach to safety prediction follows the process shown in Figure 4. For a set of input strain measurement datasets, this prediction model gives a prediction index value from 1 to 1411, which indicates a specific safety case.

### 3.3. Neural Networks

After the hierarchical classification of safety conditions under the divide-and-conquer technique, the next step is to form a deep neural network. For each level of classification discussed in the previous section, individual NN models were trained. These NN models are referred to as pre-trained NN models in this paper. There were a total of 18 pre-trained NN models (i.e., one in level I, one in level II, three in level III, and 13 in level IV), which were combined to determine a prediction model. The performances of the NN models were evaluated by computing accuracy, precision, recall, and F1 score values for each of the predicted classes, using the following equations:

(1) $$\mathrm{Accuracy}=\frac{\mathrm{TP}+\mathrm{TN}}{\mathrm{TP}+\mathrm{TN}+\mathrm{FP}+\mathrm{FN}}$$

(2) $$\mathrm{Precision}=\frac{\mathrm{TP}}{\mathrm{TP}+\mathrm{FP}}$$

(3) $$\mathrm{Recall}=\frac{\mathrm{TP}}{\mathrm{TP}+\mathrm{FN}}$$

(4) $$F1\;\mathrm{Score}=\frac{2*\mathrm{Recall}*\mathrm{Precision}}{\mathrm{Recall}+\mathrm{Precision}}$$

where true positive (TP) represents the instances in which the scaffold safety cases are correctly predicted as true, false positive (FP) represents the instances in which the scaffold safety cases are incorrectly predicted as true, true negative (TN) represents the instances in which the scaffold safety cases are correctly predicted as false, and false negative (FN) represents the instances in which the scaffold safety cases are incorrectly predicted as false.

The following sections explain the formation of the deep neural network in detail.

#### 3.3.1. Pre-Trained Neural Networks 

**Figure 5:**
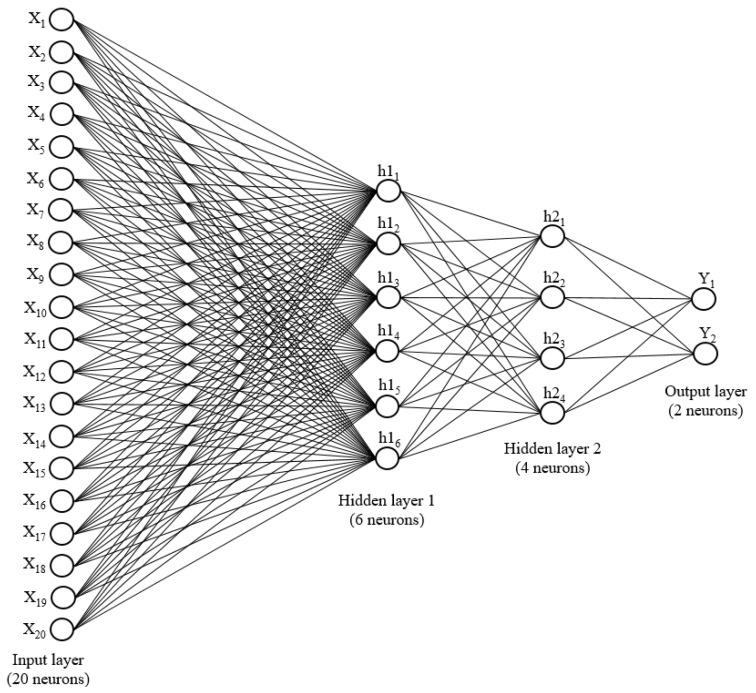
Deep neural network architecture.

A neural network typically consists of three types of layers, the first being the input layer and the last being the output layer. All layers in between are called hidden layers, and there is a minimum of one hidden layer in any network. A neural network with one hidden layer is referred to as a shallow network [32], while if the number of hidden layers is more than one, the network is referred to as a deep NN. A deep NN architecture is shown in Figure 5. The number of nodes (neurons) on the input layer depends on the number of input features used to train the NN. In this study, the number of nodes on the input layer is 20; however, the number of nodes on the output layer depends on the number of expected outputs, which is equal to the number of cases in a classification problem. The number of neurons in the hidden layer depends on the complexity of the convergence of input features [33], which means that the number of neurons increases with the complexity of the problem. Furthermore, the complexity of the convergence of input features results in additional hidden layers. An optimum number of hidden layers and nodes in each layer in the NN results in maximum prediction accuracy. Figure 5 illustrates an example of a deep NN architecture, with 20 input features and two classification cases. Accordingly, the input and output layers have 20 and 2 neurons, respectively. There are two hidden layers, with six neurons on the first and four neurons on the second. In NNs, all of the neurons in the consecutive layers are interconnected.

The design of an NN architecture is the most critical step in modeling an NN, as well as the most time-consuming, due to the requirement of multiple trials to determine the optimum number of hidden layers and nodes in each layer. The NN is continuously trained with the different number of hidden layers, consisting of different numbers of nodes, using 80% of the datasets. The remaining 20% of the datasets are used for validation purposes. To ensure better performance of the trained models, stratified five-fold validation was implemented [34], along with an early stopping technique to avoid overfitting of the trained models [35]. The process is continued with different numbers of nodes and hidden layers until the desired level of performance is achieved. For each of the pre-trained models, prediction accuracies are measured for each validation set, and the average of five validation accuracies, along with their standard deviations, are used to evaluate the trained NN models’ performance. On the basis of the precision and recall values computed for each class, it is determined whether or not there is a requirement to update the NN architecture for a particular model. For each of the pre-trained NN models, the authors selected the parameters with maximum average validation accuracy and minimum standard deviations.

#### 3.3.2. Prediction Model

The prediction model is obtained by combining all pre-trained models in sequential order, so that the model performs a stepwise analysis of the test datasets. A new database, which is different from the database used for the training and testing of the pre-trained NN models, is used to test the prediction model. The pre-trained models are loaded first, and then these models analyze all datasets from the new database to predict the safety conditions. Following these steps, the combined NN model is capable of analyzing the strain measurement datasets to predict the model scaffolds’ safety conditions out of 1411 safety cases. The performance of the prediction model was evaluated by computing precision, recall, and F1 score values for each predicted class using Equations (2)–(4), respectively. 

## 4. Results

### 4.1. Pre-Training of Neural Network Models

For pre-training the NN models, initially 1000 strain measurement datasets were used for each of the 1411 classes (total 1,411,000). While 80% of the datasets were used for training, the remaining 20% of datasets were used for testing purposes. For the first NN model with two classification cases (level I), a prediction accuracy of approximately 99% was observed. Despite a high accuracy, this may not be highly meaningful because the data proportion was unbalanced. As a result of an unbalanced proportion of datasets while implementing an NN, as discussed by Chawla et al. [36], a challenge of the incorrect classification of safe cases as unsafe was observed. In level I, the ratio of safe and unsafe categories was 1:1410; similarly, the ratio of the original number of classes on the three categories in level II was 2:385:1023. 

To overcome the minority class category misclassification, the authors increased the minority class category datasets [36] to reduce their negative impact on prediction accuracy. Additional datasets were generated to form a total of 60,000 datasets for the safe condition. Similarly, for the overturning condition in the *X*- and *Y*-directions (in level II), the authors used a total of 36,000 datasets each. For the remainder of the classes, 1000 datasets were used for each. Therefore, the new database was comprised of 1,540,000 strain measurement datasets. With the increased number of datasets for the minority class categories, the proportion of datasets in the first level of classification changed to 1:24.7, and for the second level of classification, the proportion was 1:5.3:14.2. With a new set of data, the NN models were able to make predictions for the test datasets with a minimum number of misclassifications. For the first level of classification, the entire database was used for training and testing purposes. However, for other levels of classification, the datasets corresponding to classification cases were only used during the pre-training of the NNs. Table 3 presents a summary of the NN model architecture, along with the standard deviations of the stratified five-fold validation accuracies and the average prediction accuracies for each pre-trained NN model.

### 4.2. Testing of Prediction Model

**Figure 6:**
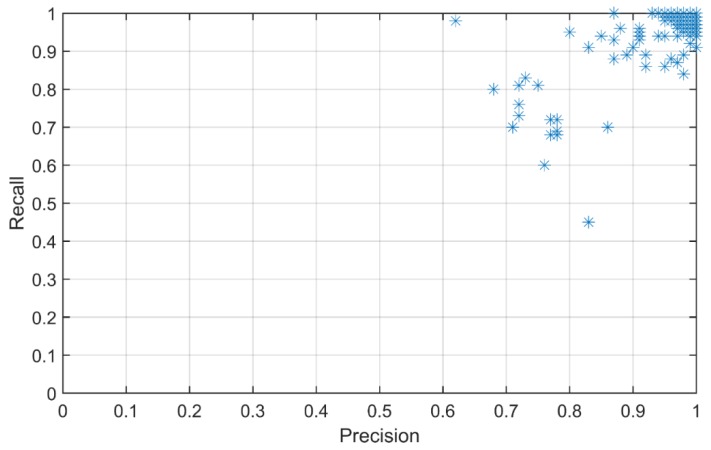
Precision–recall plot for all safety cases.

**Figure 7:**
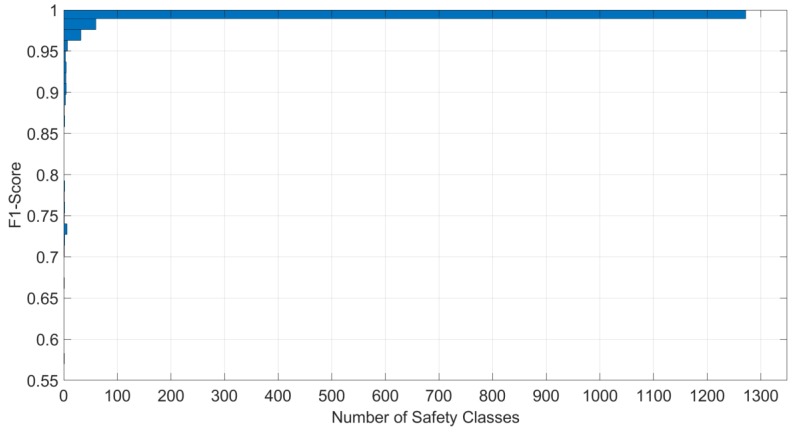
F1 score plot.

The authors used a separate strain database, consisting of 100 datasets for each of the 1411 cases, in order to test the prediction accuracy of the combined NN model. The database for testing had 141,100 strain measurement datasets (1411 cases × 100 datasets/case), each with 20 features, along with the corresponding indices for the cases. The result produced 1049 incorrect classifications (less than 1%) out of the 141,100 test datasets. Another positive observation was that none of the unsafe cases were misclassified as safe cases, which meant 100% accuracy at level I. The details of the classification results are presented in Table 4. Furthermore, Figure 6 shows the plots of recall and precision values for each of the safety classes, indicating higher values for most of the classified classes, with a few cases that are scored relatively low; the minimum precision is 0.62 and the minimum recall is 0.45. Similarly, Figure 7 is a plot of F1 scores versus the number of safety cases, which shows that the majority of classifications (i.e., 1272 out of 1411 classes) have F1 scores close to 1.00, with the minimum at a score of 0.58. The overall trend of higher F1 values indicates good performance of the prediction model.

## 5. Discussion

Initially, during the pre-training of the NN models, it was observed that highly imbalanced training data sizes (for example, 1:1410 in level I) resulted in a large number of misclassifications (i.e., incorrect classification of all safe cases into unsafe cases in level I). However, by increasing the data proportion from 1:1410 to 1:24.7, which is still an imbalanced dataset, the training results improved significantly. These results show that deep learning is powerful enough to manage such imbalanced datasets. It is also informative for discussing the proportion of misclassifications among the 1411 cases. In-depth analysis revealed that 1168 out of the 1411 cases (i.e., 82.78%) did not demonstrate any misclassifications—that is, they produced 100% accuracy. Among the remainder of the cases, only a small number of misclassifications were identified. Table 5 summarizes the number of safety cases with different numbers of misclassifications, starting from zero to more than six incorrect predictions per safety case. Most cases produced 100% accuracy; the next most accurate case produced one misclassification. Finally, there were only 32 cases (i.e., 2.27% of 1411 cases) that generated more than six incorrect classifications. 

With an increase in the number of classification classes, the complexity of training an NN increases, as it requires multiple trials with multiple hidden layers, as well as a large number of neurons in each layer, until NN parameters with the desired accuracy are obtained. Thus, the training process consumes a longer time if a single network is trained to obtain a higher classification accuracy for a very large number of cases. However, the implementation of the divide-and-conquer approach with deep learning enabled the authors to work on classifying smaller groups, providing greater flexibility to control prediction model accuracy by updating the parameters for smaller NN models whenever necessary. For instance, the higher number of incorrect classifications for some safety cases, as seen in Table 5, can be minimized by updating the parameters of the pre-trained NN models corresponding to those specific safety cases with high misclassification. As such, this approach helps overcome the challenges of large-scale classifications. Thus, such a problem-solving approach would be very beneficial for other large-scale classification problem.

## 6. Conclusions

When evaluating the safety conditions of scaffolding systems, it is essential to consider both local and global failures, as well as the complex combination of multiple member failures. The requirement to identify various member failures in a scaffold leads to a large number of failure cases, which past studies have not properly addressed. Thus, this study attempted to incorporate all potential scaffolding system failures during safety assessment, using the strain measurements of scaffold members. For a four-bay, three-story scaffold model, the authors identified 1411 safety cases. A larger database, consisting of 1,540,000 strain datasets, each with 20 strain measurement values, was used to obtain a prediction model. In order to overcome the challenges of multi-class classification using a small number of input features, this study proposed using the divide-and-conquer technique with deep learning. This allowed for performance control of the prediction model by working with smaller groups of classes at a time (i.e., breaking down a complex problem into multiple simple problems). With this approach, the authors pre-trained 18 smaller NN models, and then combined those models to form the prediction model, which demonstrated an overall prediction accuracy of 99% for the classification of 141,100 datasets corresponding to 1411 safety cases. Among the 1411 cases, the majority of cases (i.e., 82.78%) had correct classifications for all test datasets. Despite some incorrect classifications (less than 1% of the test datasets), the higher precision, recall, and F1 score values of the prediction model indicate good performance of the prediction model. 

The classification of 1411 classes, based on only 20 strain features, with such high prediction accuracy demonstrates the superiority of the proposed methodology over methods used in past scaffolding research. The divide-and-conquer approach provides the flexibility to train multiple small classification algorithms separately for different data groups, using multiple computers with low computational capacities, which reduces the computational time associated with training the deep-learning algorithm. Another benefit of using this approach is that it enables the users to improve the performance of the prediction models by updating the parameters for a smaller network, which saves a significant amount of time. Furthermore, the precise prediction results can be obtained within a fraction of a second, suggesting that the proposed methodology can be implemented reliably on an actual construction site for the real-time prediction of scaffold safety conditions, given the condition that enough data has been collected and training is completed so as to determine all of the parameters of the deep learning model in multilayers. It should be noted that this approach gives a reliable safety prediction model with the availability of a relatively small number of strain measurements. However, one drawback is the required setup and identification of the parameters of each of the smaller networks.

One of the challenges with implementation of the proposed approach lies in the initial stage of training data generation. While generating the training data, it is important to ensure that the numerical model is well designed to resemble the structural behavior of the actual structure. Without such a model, this approach cannot give a reliable prediction model, due to the lack of reliable datasets. Thus, the first requirement for the replication of this approach is to design a good model to resemble the real structure. Given the availability of training data from such a model, the methodology can be adapted for other multi-class classification problems with systems of similar complexity to the studied scaffold. Such implementation will enhance the reliability of automated safety assessment systems on construction sites. Furthermore, other construction researchers may benefit from the flexibility of working on smaller groups to solve classification problems with the divide-and-conquer technique.

## Figures and Tables

**Table 1:** Loads applied for structural analysis.

Scaffold Safety Cases	Gravity Loads (N/m^2^)	Point Loads (N)	
		*X*-Direction	*Y*-Direction
Safe	−1400 to 0	−100 to +100	−500 to +500
Over-turning	−1400 to 0	−15,000 to +15,000	−10,000 to +10,000
Unevenly settled	−1400 to 0	−100 to +100	−500 to +500
Overloading	−2000 to −1400	−100 to +100	−500 to +500

**Table 2:** Sample database of strain measurements.

**Datasets**	**Strain Measurement (µε) from Locations 1 to 10**									
	**1**	**2**	**3**	**4**	**5**	**6**	**7**	**8**	**9**	**10**
1	−7.3	−8.4	−32.3	−26.4	−6.6	−0.5	−6.3	−21.1	−8.8	3.1
2	1.7	−0.6	−11.7	−29.8	−6.5	−17.9	−1.0	−6.5	−16.5	−7.3
3	−101.2	−53.4	−39.3	−53.0	−27.0	−11.7	−63.4	−0.1	−23.4	−50.5
4	−43.4	−8.7	−37.8	−90.1	−95.2	−80.8	−86.5	−95.5	−77.7	−93.6
5	−1.2	−55.5	−15.7	−91.8	−76.9	−103.8	−96.9	−84.4	−86.9	−77.3
6	0.1	−31.9	−10.9	−6.5	−2.5	−26.9	−20.1	0.4	−35.4	−11.5
**Datasets**	**Strain Measurement (µε) from Locations 11 to 20**									
	**11**	**12**	**13**	**14**	**15**	**16**	**17**	**18**	**19**	**20**
1	−1.0	−9.6	−2.7	−13.1	−7.3	−0.1	−7.1	−12.8	−10.0	3.1
2	3.1	−0.2	−1.4	−0.1	−0.4	−0.6	−0.5	−9.1	−0.5	−2.3
3	−11.1	−47.0	−27.0	−27.5	−14.6	−10.0	−48.8	−0.1	−20.7	−45.0
4	−31.7	−6.5	−37.3	−49.7	−5.5	−18.5	−28.5	−26.6	−22.7	−27.4
5	−1.2	−54.6	−13.4	−5.5	−24.6	−7.3	−38.9	−41.9	−21.6	−29.2
6	0.1	−15.7	−10.1	−5.5	−3.1	−7.5	−0.8	−1.3	−23.0	−7.3

**Table 3:** Pre-trained neural network (NN) model architecture and pre-training results.

Level	Model No.	No. of Classes	NN Architecture	Stratified Five-Fold Validation Accuracies	
				Standard Deviation of Accuracies	Avg. Accuracy
I	1	2	55, 40	±0.00%	99.99%
II	2	3	60, 50, 40	±0.00%	97.61%
III	3	2	60, 50, 40	±0.00%	100.00%
III	4	4	80, 60, 50, 20	±0.03%	99.48%
III	5	10	100, 80, 60, 40, 25	±0.21%	99.86%
IV	6	10	80, 60, 50, 20	±0.07%	99.74%
IV	7	45	80, 60, 50, 20	±0.04%	99.71%
IV	8	120	80, 60, 50, 40, 20	±0.17%	97.53%
IV	9	210	100, 80, 50, 40, 25	±0.04%	97.16%
IV	10	10	80, 60, 50, 20	±0.06%	99.91%
IV	11	45	80, 60, 50, 20	±0.01%	99.97%
IV	12	120	80, 60, 50, 20	±0.00%	99.99%
IV	13	210	80, 60, 50, 20	±0.04%	99.98%
IV	14	252	80, 60, 50, 20	±0.10%	99.93%
IV	15	210	80, 60, 50, 20	±0.04%	99.96%
IV	16	120	80, 60, 50, 20	±0.02%	99.99%
IV	17	45	80, 60, 50, 20	±0.02%	99.98%
IV	18	10	60, 40, 20	±0.02%	99.99%

**Table 4:** Summary of final classification results.

Total number of safety condition cases	1411
Total number of test datasets	141,100 (i.e., 100 datasets for each class)
Prediction accuracy	99.26%
Total number of incorrect classifications	1049 out of 141,100 (i.e., 0.74%)
Range of misclassifications	0 to 55 out of 100 datasets for each class

**Table 5:** Summary of misclassifications for different cases.

Number of Misclassifications Per Safety Cases (Out of 100)	Number of Safety Cases	Proportion of Misclassified Safety Cases (%)
0	1168	82.78
1	111	7.87
2	46	3.26
3	24	1.70
4	14	0.99
5	8	0.57
6	8	0.57
>6	32	2.27
